# Chinese herbal medicine for immune infertility: A protocol for systematic review and meta-analysis

**DOI:** 10.1097/MD.0000000000024248

**Published:** 2021-02-05

**Authors:** Cui Jiang, Zhaodi Wang, Shiqing Yuan, Yong Jiang, Ying Ye

**Affiliations:** School of Basic Medical Sciences, Chengdu University of Traditional Chinese Medicine.

**Keywords:** Chinese herbal medicine, effectiveness, immune infertility, meta-analysis, protocol, safety

## Abstract

**Background:**

Infertility is a reproductive disorder caused by multiple causes and is an adverse event of reproductive health for couples in the reproductive period. Women who do not avoid sex for at least 12 months and are not pregnant are said to be infertile. 10% to 20% of infertility is caused by immune factors. At present, there is no unified diagnostic standard for immunological infertility. Clinically, it is considered that abnormal ovulation and reproductive system function of women are excluded, and no obvious pathogenic factors occur; routine examination of male semen is normal, but there is evidence of anti-reproductive immunity, thus causing infertility is immunological infertility. Traditional Chinese medicine (TCM) has a long history of treating infertility and has remarkable curative effect. It plays an important role in the treatment of gynecological and obstetrical diseases in China. The purpose of this study is to evaluate the efficacy and safety of traditional Chinese medicine for the treatment of immune infertility.

**Method:**

we searched the literature from following databases: Cochrane Library, PubMed, China Biomedical Literature Database (CB), EMBASE, Chinese Journal of Science and Technology (VIP), China National Knowledge Infrastructure Database (CNKI) and Wanfang Database were searched. All the databases mentioned above will be searched from the start date to the latest version. A manual search of all references to the included trials, published randomized controlled trials (RCTs) whether blind or unblind, any languages and length of follow up were included. Treatments included Chinese medicinal herbs (single or compound). Controlls were placebo and western medicine, or no intervention. Key outcomes will include pregnancy rates, the efficiency of Chinese herbal medicine (at least one negative antibody for infertility), birth rates (the ratio of the number of pregnant women giving birth to their babies normally after herbal treatment to the total number of patients treated), recurrence rate and safety index. Two evaluators independently retrieved and extracted data and import it into Endnote X8. Then they conduct methodological evaluation on the quality of the included studies, and meta-analysis was conducted with RevMan 5.3 and Stata 13.0 software. We will use the Cochrane risk analysis tool to assess the risk of bias. Differences will be resolved by consensus or through the participation of third parties. All analysis will be performed based on the Cochrane Handbook for Systematic Reviews of Interventions.

**Results:**

The purpose of this study is to evaluate the efficacy and safety of traditional Chinese herb medicine in the treatment of immune infertility.

**Conclusion:**

This meta-analysis can provide evidence for clinicians to help patients make better choices.

**Trial registration number:**

INPLASY2020120073.

## 1 Introduction

Ten percent and twenty percent of infertility is caused by immune factors.^[1,2]^ Clinically, it is considered that if abnormal ovulation and reproductive system function of women are excluded, there is no obvious pathogenic factor, and there is no abnormal in routine examination of male semen, but there is evidence of anti-reproductive immunity, thus causing infertility is immune infertility.^[3]^ Studies have shown that immunological infertility is associated with a variety of reproductive immune antibodies, including anti-sperm antibody (As Ab), anti-endometrial antibody (Em Ab), anti-cardiolipin antibody (Ac Ab), anti-egg cell antibody (AOAb), anti-Permeability belt antibody (ZPAb), and anti-chorionic gonotropin antibody (Ah CGAb), etc.^[4,5]^ Anti-sperm antibody is a complex pathological product. If spermatic immune factor deficiency or reproductive tract enzyme deficiency, sperm can be used as antigen to stimulate the body to produce antisperm antibody, which can affect sperm forward movement through circulation to cervical mucus, resulting in infertility.^[6]^ In patients with endometriosis or endometritis, the positive rate of anti endometrial antibody was significantly increased. The antibody reacts with endometrium and activates the immune system to damage endometrium and oviduct tissue, which affects the transport of oviduct and the biochemical metabolism and physiological function of endometrial tissue cells, and affects embryo implantation and development.^[7–10]^ Abnormal autoimmune function or IgG-like globulin such as anti FSH antibody in the body can cause the destruction of germ cells in the ovary, accelerate follicular atresia, and affect follicular development and ovulation.^[11]^ HCG can be inactivated by anti hCG antibody, which can not maintain early pregnancy, leading to abortion, HCG in chorionic tissue of women with history of spontaneous abortion, induced abortion and biochemical pregnancy may act as antigen to stimulate the mother to produce antibody.^[6]^ Anticardiolipin antibodies combine with cardiolipin on the trophoblast surface, resulting in cell damage, activation of platelets and platelet aggregation, which cause decidual vascular lesions as well as placental thrombosis, infarction and damage to placental function, so that it can not be transferred from biochemical pregnancy to clinical pregnancy.^[12,13]^ The mechanism of anti zona pellucida antibody production is still unclear, It is speculated that zona pellucida is absorbed repeatedly after ovulation and follicular atresia. The cross antigen with zona pellucida or various pathogenic factors can cause structural deformation of zona pellucida protein, as well as immune recognition dysfunction in vivo, which can stimulate the body to produce anti zona pellucida antibodies that will affect fertilization and egg implantation.^[14]^ When the body has anti-trophoblast antibody, the mother cannot induce the production of blocking antibody. When the embryo is implanted, the mother will produce immune response to attack the embryo, resulting in abortion.^[15]^ It can be seen that the production of immune response can affect follicular development, ovulation, fertilization, fertilized egg implantation and placental function, and eventually lead to infertility.^[16]^ Modern medicine has a thorough study on the etiology of immunological infertility. At present, western medicine mainly treats immunological infertility through immunosuppressive drugs, anticoagulants, isolation therapy and assisted reproduction.^[17–20]^ However, there are some problems: such as long-term side effects of immunosuppressive therapy, high cost of assisted reproduction therapy and low success rate. Traditional Chinese medicine (TCM) has a long history of treating infertility, which is effective and relatively safe. Studies show that traditional Chinese medicine and conventional western medicine in the treatment of immune infertility in the rate of antibody conversion, pregnancy rate and recurrence rate are better than the simple application of western medicine in the treatment of this disease.^[21–23]^ In recent years, there have been more and more studies on the treatment of immune infertility with Traditional Chinese medicine.^[24,25]^ However, further studies are needed to evaluate the efficacy and safety of traditional Chinese medicine in the treatment of immunological infertility. Therefore, our aim is to collect the latest data on the treatment of immunological infertility by TCM and evaluate its efficacy and safety to support clinical decision-making.

## 2 Methods and analysis

### 2.1 Study registration

This systematic review protocol has been registered on INPLASY as INPLASY2020120073. All of the data for the article have been published online, so this protocol does not require ethical approval. The protocol will be strictly enforced according the Guide to the contents of a Cochrane protocol and review (Part 1. Chapter 4 of Cochrane Handbook for systematic review of interventions Version 5.1.0).

### 2.2 Inclusion criteria for study selection

#### 2.2.1 Types of studies

We will search all the studies that TCM is used as the main intervention for immune infertility, Non-RCTs quasi-RCTs, series of case reports, and cross research will be excluded. Full article not available will be excluded. No language restrictions.

#### 2.2.2 Types of participants

All the patients who have been diagnosed with immune infertility will be included. There are no restrictions on age, region, nation, belief, ethnicity, sources, and courses of disease.

#### 2.2.3 Types of interventions

There is no requirement for the intervention course, the specific contents of the control group and the experimental group are as follows.

##### 2.2.3.1 Control intervention

The control group received routine western medicine treatment, prednisone plus aspirin.

##### 2.2.3.2 Experimental interventional

The experimental group is treated with TCM on the basis of conventional western medicine treatment in the control group. The use of TCM is limited to prescription and Chinese patent medicines. Prescription drugs require a clear dose, but there are no restrictions on the composition, dosage form and dosage. For the dosage, such as decoctions, granules, pills, powders, etc. Other types of TCM treatments such as TCM injections, acupuncture, moxibustion, massage, cupping, and others will be excluded.

### 2.3 Outcome measures

#### 2.3.1 Primary outcomes

The primary outcomes are pregnancy rates, the efficiency of Chinese herbal medicine (at least one negative antibody for infertility) and safety index.

#### 2.3.2 Secondary results

The secondary evaluation criteria were as follows: birth rates (the ratio of the number of pregnant women giving birth to their babies normally after herbal treatment to the total number of patients treated) and recurrence rate.

### 2.4 Exclusion criteria for study selection

1. The studies without primary outcomes;
2. the studies that cannot be obtained in full text or no data can be extracted;
3. the studies with obvious errors in the data;
4. the one with the higher cases will be selected, while the studies have the same data source as the cases;
5. for duplicate publications or similar studies, just select one of them.

### 2.5 Searching strategy

#### 2.5.1 Electronic searches

The databases of PubMed, CENTRAL (The Cochrane Central Register of Controlled Trials), Excerpt Medica Database (Embase), China National Knowledge Infrastructure (CNKI), Weipu Information Chinese Periodical Service Platform (VIP), Wanfang Data Knowledge Service Platform (WANFANG Data), and China Biomedical Literature Service System (SinoMed) will be searched online. The search time is set from the establishment of the search database to December 2020. According to the standards of the Cochrane Collaboration workbook of the International Evidence-Based Medicine Center, the search terms include “Chinese medicine,” “Traditional Chinese medicine,” “proprietary Chinese medicine,” “Chinese herbal medicine,” “immune infertility.” The complete PubMed search strategy is summarized in Table 1.

**Table 1:** Search strategy used in PubMed database.

Number	Search termse
1	Search (Traditional Chinese Medicine) OR (TCM) OR (Traditional Medicine, Chinese) OR (Zhong Yi Xue) OR (Chinese Traditional Medicine) OR (Chinese Medicine) OR Chinese Herb Medicine) OR (Chinese Herb)
2	Search: (Immune Systems) OR (System, Immune) OR (Systems, Immune)OR (Systems, Immune)
3	Search (Randomized controlled trial)
4	1 and 2 and 3

### 2.6 Data collection and analysis

#### 2.6.1 Study description

**Figure 1:**
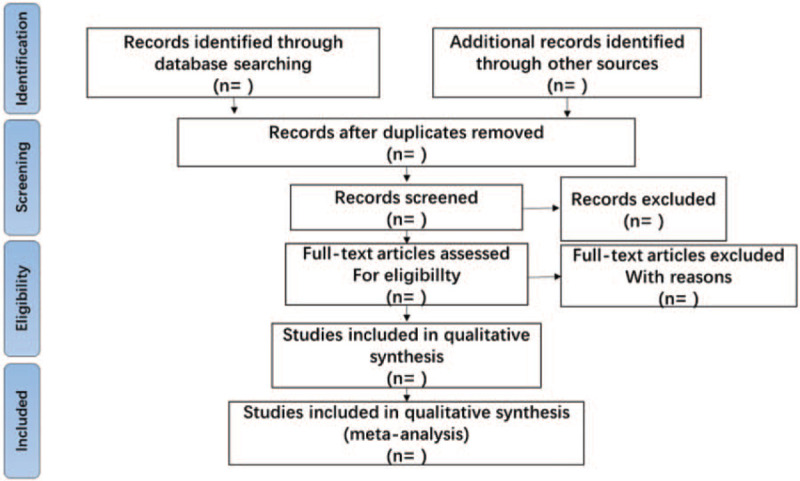
The PRISMA flow chart.

1. The initial screening: Retrieve several databases through the above search terms, and then import potentially relevant literature into Endnote X8. Researchers will use Endnote X8 to check duplicates and eliminate duplicate literature. Roughly browse documents to prevent careless omissions of the software.
2. The 2nd time of screening the literature: Two researchers will browse titles and abstracts based on inclusion and exclusion criteria, so as to exclude documents that are not related to the research, such as case reports, animal experiments, theoretical researches, studies on non- immune infertility. The details of selection process will be shown in the PRISMA flow chart in Figure 1.
3. The 3rd time of screening the literature: Carefully reading the remaining documents and strictly filtering out unqualified documents such as general controlled trials, lacking control group, deficiency of random allocation, incompatible outcome indicator, the appearance of similar data, etc.

As for the literature that cannot be ensured, it would be confirmed by the discussion of the 2 researchers. If they encounter disagreement, the third researcher will resolve it through consultation or questioning. For documents where full text is not available, contact the corresponding author for full text. Read the full-text to determine whether to include it.

#### 2.6.2 Data extraction and management

First of all, a unified data extraction form will be developed. The data extraction of the literature is also independently completed by 2 researchers. This process can be completed with the help of excel software. The data extraction form is roughly as follows:

1. Basic information: article title, all authors, publication time, contact information. Article number uses first authors last name plus abbreviation of first name plus year of publication time, for example, related papers published by John Wilson in May 2020 will be recorded as Wilson J. 2020.
2. Research methods: diagnostic criteria, efficacy evaluation criteria, test type, sample size, generation of random number series, implementation of blind method, allocation concealment.
3. Participants: patients age, course of disease, location, number of cases in the control and experimental group.
4. Intervention method: Chinese herbal medicine, period of treatment, treatment frequency.
5. Outcome measures: primary observation indicators, secondary observation indicators, improvement of indicators before and after treatment, detailed records of adverse reactions.
6. Source of funding and medical ethics review.
7. Others: number and reasons of dropouts or lost follow-up during the trial, etc.

#### 2.6.3 Risk of bias evaluation

As for the risk of bias in the literature, 2 researchers will independently use the tool for assessing risk of bias recommended by Cochrane Handbook for Systematic Reviews of Interventions 5.1.0 (Cochrane Handbook 5.1.0—Part 2: 8.5–8.7) to assess the quality of the included literature and risk of bias. Evaluation content includes: selection bias (random sequence generation, and allocation concealment), performance bias (blinding of participants and personnel), detection bias (blinding of outcome assessment), attrition bias (incomplete outcome data), reporting bias (selective outcome reporting), and other bias (other sources of bias). Evaluators judge the risk level by carefully reading the full text, which is divided into low risk, high risk and unknown risk. If the research reported in the literature is not detailed enough, the judgement is usually “unknown risk” of bias. For example, the study uses random number table for grouping, so the random sequence generation will be expressed as “low risk.” If there are any differences, we would consult the third reviewer for solution.

#### 2.6.4 Statistical analysis

The meta-analysis in this review will use RevMan 5.3 and Stata 13.0 software. For the outcome index of the 2 categorical variables, relative risk (Relative risk, RR) will be adopted, and for the outcome index of continuous variables, the mean difference (MD) or standardized mean difference (SMD) will be adopted will a confidence interval (CI) of 95%. Heterogeneity tests will be used for the included studies which will be tested by Chi-Squared test. If *P* ≥ .10 and *I*^2^ ≤ 50%, there is no significant statistical heterogeneity or no statistical difference in heterogeneity, a fixed effect model will be adopted. If *P* < 10 and/or *I*^2^ > 50%, there is significant heterogeneity between studies, a random effect model will be adopted. Further analysis of the source of heterogeneity, if necessary, perform subgroup analysis. There are clinical and methodological differences in the experimental studies. Therefore, random effects models will be selected in this study. Finally, a funnel chart will be drawn to evaluate the publication bias of the literature. Sensitivity analysis will be performed to test the robustness of findings if there are sufficient studies included. We will conduct sensitivity analysis by excluding

1. studies with high risks of bias and
2. outliers that are numerically distant from the rest of the data.

#### 2.6.5 Publication bias

If a result of a meta-analysis contains more than 10 articles and above, we will use a funnel plot to test whether there is a publication bias. If the number of articles included in the study is <10, the publication bias is not significant.

#### 2.6.6 Quality of evidence

The quality of evidence will be assessed by Grades of Recommendations Assessment, Development and Evaluation (GRADE). The evaluation included: downgrade quality of evidence (risk of bias; inconsistency; indirectness; imprecision; publication bias) and upgrade quality of evidence (large effect; plausible confounding; dose–response gradient). The quality of evidence will be divided into 4 levels: high, moderate, low, and very low. Finally, refine the data and use software to edit, analyze, and draw summary of findings table.

## 3 Discussion

In recent years, the clinical RCT of immune infertility is on the rise.^[26–32]^ However, clinicians have not reached a consensus on treatment principles and lack for a unified standardized standard for the treatment of this disease. Western medicine treatment for this disease has some limitations, treatment results are not satisfied. Chinese medicine can help make up for the shortcomings of current treatments and improve clinical efficacy. Traditional Chinese Medicine has a profound theoretical basis and rich clinical experience in the treatment of infertility. TCM treat this disease through tonifying the kidney and spleen, soothing the liver and promoting blood circulation to harmonize Qi and blood, and balance yin and Yang. A considerable number of clinical studies have shown that traditional Chinese medicine can improve the negative rate of antibody and pregnancy rate. Therefore, we will evaluate the efficacy and safety of traditional Chinese medicine in the treatment of immune infertility. The results of this study can provide a possible ranking for TCM treatment of immune infertility. We hope that these results can provide reference for clinicians in the treatment of immune infertility and help doctors make the best clinical decisions.

## Author contributions

**Conceptualization:** Cui Jiang, Ying Ye.

**Data curation:** Cui Jiang, Zhaodi Wang, Shiqing Yuan.

**Formal analysis:** Cui Jiang, Zhaodi Wang, Shiqing Yuan, Ying Ye.

**Funding acquisition:** Ying Ye.

**Methodology:** Cui Jiang, Zhaodi Wang, Shiqing Yuan.

**Project administration:** Cui Jiang, Yong Jiang.

**Supervision:** Ying Ye.

**Writing – original draft:** Cui Jiang, Shiqing Yuan, Ying Ye.

**Writing – review & editing:** Cui Jiang, Shiqing Yuan, Ying Ye.
